# The Euvichol story – Development and licensure of a safe, effective and affordable oral cholera vaccine through global public private partnerships

**DOI:** 10.1016/j.vaccine.2018.09.026

**Published:** 2018-10-29

**Authors:** Lina Odevall, Deborah Hong, Laura Digilio, Sushant Sahastrabuddhe, Vittal Mogasale, Yeongok Baik, Seukkeun Choi, Jerome H. Kim, Julia Lynch

**Affiliations:** a Life Science Consultant, Gothenburg, Sweden; b International Vaccine Institute, Seoul, Republic of Korea; c Médecins Sans Frontières, Seoul, Republic of Korea; d EuBiologics Co., Ltd, Seoul, Republic of Korea

**Keywords:** Cholera, Oral cholera vaccines, Neglected diseases, Public-private partnerships, Infection prevention and control

## Abstract

Cholera, a diarrheal disease primarily affecting vulnerable populations in developing countries, is estimated to cause disease in more than 2.5 million people and kill almost 100,000 annually. An oral cholera vaccine (OCV) has been available globally since 2001; the demand for this vaccine from affected countries has however been very low, due to various factors including vaccine price and mode of administration. The low demand for the vaccine and limited commercial incentives to invest in research and development of vaccines for developing country markets has kept the global supply of OCVs down. Since 1999, the International Vaccine Institute has been committed to make safe, effective and affordable OCVs accessible. Through a variety of partnerships with collaborators in Sweden, Vietnam, India and South Korea, and with public and private funding, IVI facilitated development and production of two affordable and WHO-prequalified OCVs and together with other stakeholders accelerated the introduction of these vaccines for the global public-sector market.

## 1 Introduction

Vaccines are among the most cost-effective public health interventions, contributing significantly to the reduction of mortality and morbidity from infectious diseases in industrialized countries in the 20th century. Still the World Health Organization (WHO) estimates 1.5 million children under the age of five die annually from infectious diseases that could have been prevented by vaccination [1].

Several obstacles keep vaccines from reaching people in need. Among these; (1) *Perceived lack of profitable markets by vaccine producers*. Because of the high costs and risks associated with vaccine development, a high return on investment is required by manufacturing companies, making vaccines targeting diseases mainly found in low- and middle-income countries (LMICs) unattractive for development as the demand for vaccines does not always coincide with the ability to pay; (2) *No introduction of available vaccines due to uncertainty.* Information on disease burden, cost of illness and vaccination cost effectiveness analysis are often limited in developing country settings, making decision-making and priority-setting difficult leading to low demand by affected countries even though vaccines are needed; and (3) *Available vaccines not made for use in developing countries.* Many vaccine administration regimens are complicated, making vaccination programmatically challenging in resource-limited settings.

A vaccine against cholera is an example of a vaccine that was facing these challenges. Cholera is an acute, rapidly-dehydrating diarrheal disease transmitted through water or food contaminated with the bacteria *Vibrio cholerae O1* and *O139*. It is a disease of poverty primarily affecting people living in areas with difficult access to clean drinking water, and inadequate hygiene and sanitation [2]. The incubation period is between 12 h and five days and if not treated properly, a cholera infection can lead to death within hours.

Cholera occurs both as endemic disease and in outbreaks, which can include large, explosive epidemics. The global burden of cholera is not fully known because of under-reporting, with some affected countries not reporting cases at all to avoid the stigma often associated with the disease and its economic impact. In 2015, a total of 172,454 cholera cases and 1304 deaths were reported to the World Health Organization (WHO) by 42 countries [2], but this number is likely a significant underestimation. A recent study estimates approximately 1.3 billion people are at risk for cholera in a total of 69 endemic countries, and 2.86 million cholera cases occur annually in these countries. Among these cases, there are an estimated 95,000 deaths. Most of the global burden is in sub-Saharan Africa (60%) and South-East Asia (29%) [3].

The first vaccine against cholera, a whole-cell (WC) injectable vaccine, was developed in 1885. Although several additional injectable cholera vaccines were developed in the late 19th and early 20th century, they were shown to be reactogenic with limited efficacy and unsuitable for large scale public health programs. Vaccination against cholera was eventually removed by the WHO from recommended cholera-control measures in 1973 [4]. It was not until 2001 that the first oral cholera vaccine (OCV), developed at the University of Gothenburg, Sweden and licensed in 1991 (Dukoral®, Crucell) [5], [6] was prequalified by WHO, enabling it for purchase by United Nations procurement agencies.

**Fig. 1:**
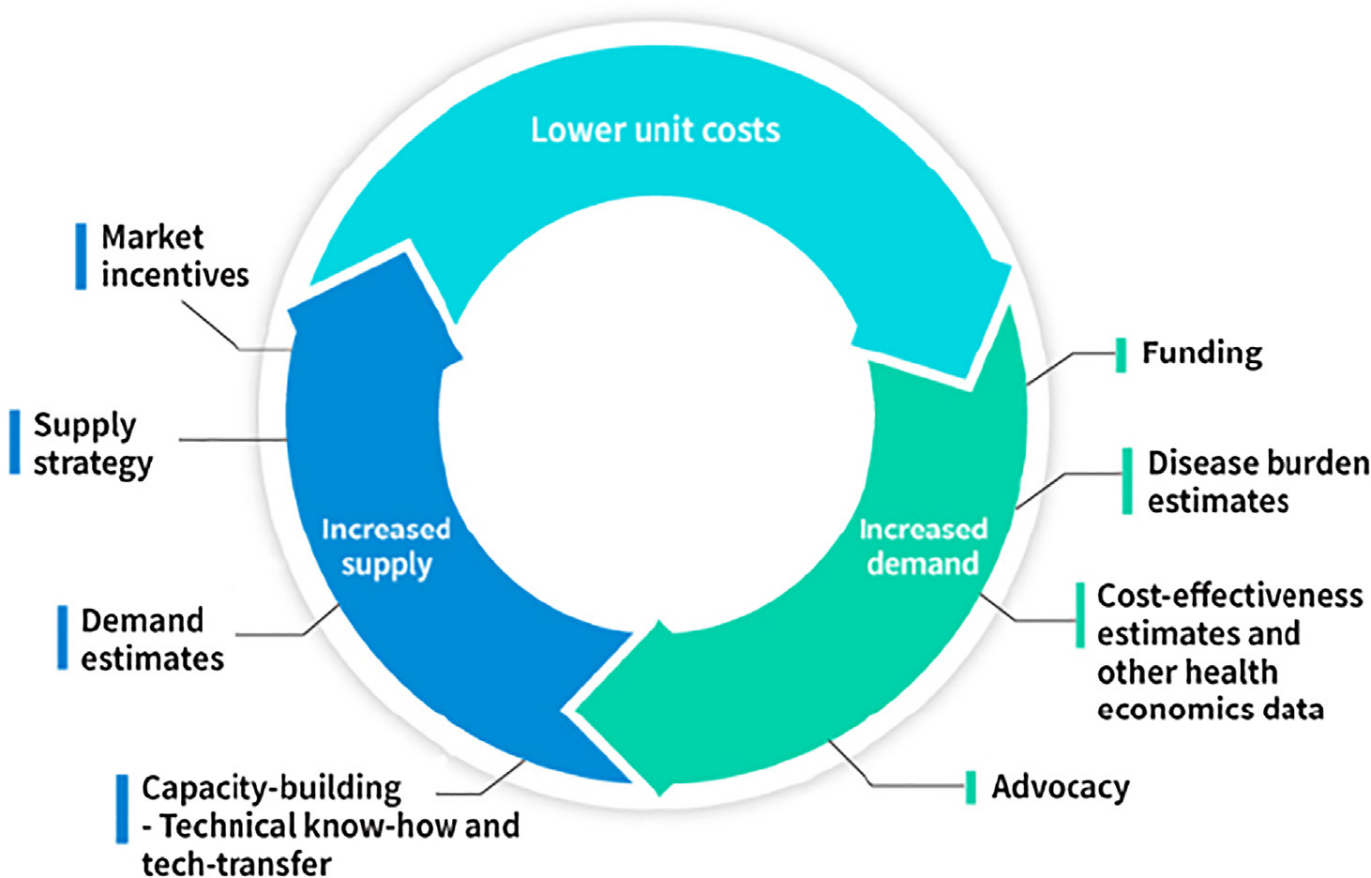
Desired virtuous cycle of supply-demand for cholera vaccines. A virtuous cycle of supply-demand for cholera vaccines can be reached by an increased demand (achieved through for example disease burden estimates and advocacy) which in turns leads to current producers increasing their production capacity or new producers entering the market. This results in a growing global production capacity which in turn can lead to lower prices and distribution of vaccine to more people. Figure inspired by Milstein et al. [58].

However, despite the availability of a WHO-prequalified cholera vaccine, OCV demand remained low. The main reasons for low demand were probably the price (one dose of Dukoral® costs about $6 to the public sector), and the requirement of coadministration with buffer as the vaccine contains a recombinant cholera toxin B subunit sensitive to the acidic environment in the stomach. While Dukoral has been used in emergency situations and in demonstration projects in endemic areas [7], the vaccine remained mainly a travelers’ vaccine, used by people from industrialized countries going to cholera-endemic areas and not accessible to populations really in need. In spite of the high burden of cholera disease, the relatively low demand for OCVs has made producers hesitant to invest in the production of cholera vaccines which long kept cholera vaccines in a “vicious cycle” of high unit costs and inadequate supply for the public-sector market. To break this vicious cycle and create a “virtuous cycle” with increased demand and production and lower unit costs (Fig. 1), the International Vaccine Institute (IVI) initiated efforts to develop a safe, efficacious and affordable cholera vaccine in 1999.

*In this paper, we describe IVIs efforts, through a variety of partnerships with collaborators in Sweden, Vietnam, India and South Korea and with support from public and private funding, to increase the supply of affordable OCVs and working together with many other stakeholders shape the demand for affordable oral cholera vaccines globally. With a focus on IVI’s partnership with the South Korean company Eubiologics, we describe the important role of product development partnerships to facilitate the development and production of a vaccine for a neglected disease like cholera. By mobilizing resources and political will, creating commercial incentives, and building public- and private-sector partnerships, IVI working with others helped remove several of the obstacles limiting vaccine development and production for this neglected disease.*

### 1.1 The International Vaccine Institute and the Cholera Vaccine Program

The International Vaccine Institute is a not-for-profit international organization, funded through governmental and philanthropic support, with the mission to discover, develop and deliver safe, effective and affordable vaccines for global public health. To help fulfill its mission, IVI uses a three-component approach: (1) *Research* – vaccine research and development, translational and field research; (2) *Partnerships* – product development partnerships, international research consortia, and networks; and (3) *Capacity building* – training, technical assistance, and technology transfer.

Through the Cholera Vaccine Program, IVI aims to reduce the global disease burden of cholera by accelerating the development and delivery of affordable oral cholera vaccines against epidemic and endemic cholera, and to make them accessible to populations at risk.

**Fig. 2:**
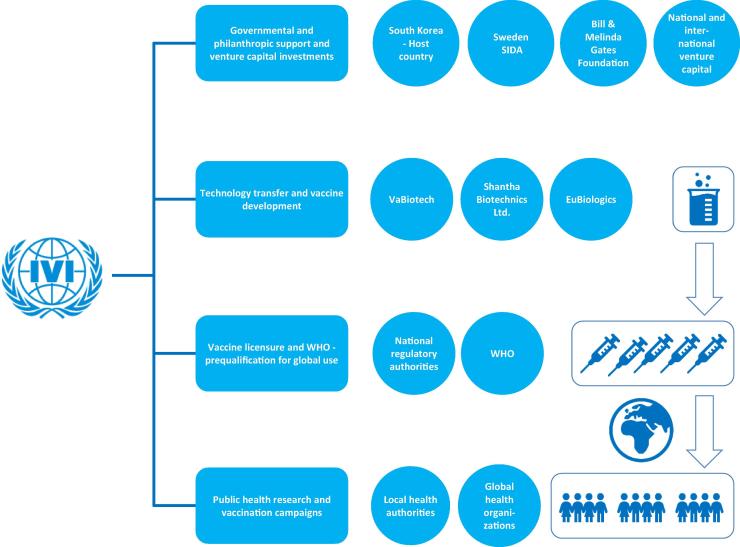
The Cholera Vaccine Program. Through partnership with funders, private sector, NGOs and public-sector IVI can catalyze the development, production and licensure of affordable oral cholera vaccines and accelerate the introduction of theses vaccines to the global market. This figure represents the major partners and steps (described in blue boxes and circles) in the Cholera Vaccine Program. (For interpretation of the references to colour in this figure legend, the reader is referred to the web version of this article.)

For a schematic description, showing the major partners and collaborating organizations and steps of the Cholera Vaccine Program and the overall time line of the program, please see Fig. 2 and Table 1*.*

**Table 1:** Timeline of major milestones in the development of Cholera vaccines.

*1894–1960* • Development of several injectable whole-cell cholera vaccines
*1980s* • Monovalent killed whole-cell oral cholera vaccine developed at University of Gothenburg, Sweden • Technology transfer from University of Gothenburg to Vietnam and production of oral cholera vaccine in Vietnam
*1991* • Licensure of monovalent killed whole-cell oral cholera vaccine developed at University of Gothenburg (Dukoral®); Dukoral mainly marketed as travelers’ vaccine
*1997* • Reformulation and production of the Vietnamese vaccine by VaBiotech. The vaccine is licensed as ORC-Vax™ in Vietnam
*Early 2000s* • Initiation of partnership between IVI and VaBiotech • Reformulation of the VaBiotech vaccine in collaboration with IVI
*2001* • WHO prequalification of Dukoral®
*2002–2005* • Clinical trials of the IVI-reformulated vaccine in Vietnam and India demonstrate vaccine is safe and immunogenic • Initiation of partnership between IVI and Shantha Biotechnics Ltd., in India • Technology transfer from VaBiotech to Shantha Biotechnics Ltd
*2006* • Cluster-randomized, placebo-controlled efficacy trial with IVI-reformulated vaccine in Kolkata, India demonstrate that the vaccine is safe and efficacious (65%)
*2009* • Licensure of IVI-reformulated vaccine in Vietnam (mORCVAX™) • Licensure of IVI-reformulated vaccine in India (Shanchol®)
*2010* • WHO Position Paper recommends OCV to be used in endemic areas and to be considered for use in areas at risk for outbreaks • Initiation of partnership between IVI and EuBiologics in South Korea • Laboratory-scale technology transfer (3L) from IVI to EuBiologics
*2011* • WHO prequalification of Shanchol™ making it the first low-cost OCV available for the global market • EuBiologics produce Euvichol® in 100L fermenter • Pre-clinical studies of Euvichol
*2012* • Phase I clinical study of Euvichol® in Korea
*2013* • A global cholera vaccine stockpile is created • Gavi supports stockpile
*2014* • WHO recommendation of OCV in humanitarian emergencies • Clinical studies of Euvichol® in the Philippines demonstrate vaccine is safe and efficacious • Financing for Euvichol® development and production scale-up to EuBiologics by investors • Production capacity scale-up (100L to 600L fermenter) by EuBiologics increases Euvichol® production to up to 25 million doses per year
*2015* • OCV demand surpasses global supply • WHO prequalification of Euvichol®
*2016* • WHO prequalification of 600L scale (No thimerosal) Euvichol variation • Long term arrangement with UNICEF signed for Euvichol
*2017* • WHO prequalification of Euvichol-Plus®

### 1.2 Development of an affordable WHO-prequalified OCV through collaboration with vaccine producers in Vietnam and India

The first step towards a safe, efficacious and affordable cholera vaccine for global use was taken through a product development partnership (PDP) between IVI and the Company for Vaccine and Biological Production No.1 (VaBiotech), a vaccine manufacturer under the Ministry of Health of Vietnam. Since the mid-1980s, Vietnam had been producing a low-cost killed monovalent O1 whole-cell oral cholera vaccine that did not contain the cholera toxin B subunit and hence did not require a buffer (making if more amenable to use in resource-limited settings) for the country’s public health programs [8], [9]. To protect from the emergence of *Vibrio cholerae* O139 seen in Bangladesh and India in the early 1990s this vaccine was reformulated into a bivalent vaccine by addition of O139 and further produced and licensed by VaBiotech as ORC-Vax™ in 1997. ORC-Vax™ was proven safe and effective in the Vietnamese population [10] however because the Vietnamese national regulatory authority (NRA) was not formally certified by the WHO as meeting all indicators for a functional vaccine regulatory system at that time, the vaccine could not be prequalified and procured by UN agencies for global distribution. To ensure the vaccine met international Good Manufacturing Practice (GMP) standards and WHO production guidelines for global use IVI partnered with VaBiotech to reformulate the vaccine. Through the partnership the strain composition was changed by replacing a high toxin-producing strain (classical Inaba 569B) to two alternative strains: heat-killed classical Inaba Cairo 48 and formalin-killed classical Ogawa Cairo 50 and the dose of the O1 Ogawa component was increased. In addition, new lot release assays were developed that provided greater consistency in the product formulation and that better detected the removal of cholera toxin. With these changes a vaccine with a higher yield, lower production costs and hence a more affordable price was developed. The reformulated low-cost vaccine was tested in clinical trials in both Vietnam and India and was demonstrated to be safe [11] and more immunogenic than the earlier version of ORC-Vax [12].

To ensure the reformulated vaccine could be made available internationally, IVI facilitated a technology transfer between VaBiotech and Shantha Biotechnics Ltd., a private biotech company in Hyderabad, India (acquired by Sanofi in 2009), a country with a national regulatory authority certified as fully functional by WHO. In parallel with the technology transfer, IVI, in collaboration with India’s National Institute of Cholera and Enteric Diseases (NICED), conducted a cluster-randomized, placebo-controlled efficacy trial with the reformulated vaccine in Kolkata, India. The two-dose vaccine was shown to provide a protective efficacy of 65% for at least 5 years [13], [14], [15] and this was the first ever demonstration of strong sustained protection by an oral cholera vaccine. In 2009, the vaccine was licensed in both Vietnam (mORCVAX™) and India (Shanchol®), and in 2011, Shanchol® was approved by the WHO Prequalification Program, making it the first affordable OCV ($1.85 per dose) available for the global public market.

### 1.3 The need for increased supply of low-cost cholera vaccines

Concerns about cholera increased steadily during the early 2000 as a result of the increased frequency of large and often protracted cholera epidemics in sub-Saharan Africa and Asia and the emergence of new, more virulent, strains of *V. cholerae O1*. These public health concerns, as well as the availability of safe and effective OCVs [16], [17], [18], [11], prompted WHO to issue a Position Paper on Cholera Vaccines in March 2010. The WHO position paper, which provides guidance to member states on health policy, stated that “OCV should be used in endemic areas and should be considered for use in areas at risk for outbreaks in conjunction with other prevention and control strategies” [19]. Several major cholera outbreaks in 2010, including the devastating outbreak in Haiti with over 8000 deaths, further contributed to raising interest in the oral cholera vaccine by the international community and affected countries. The possibility of establishing an OCV stockpile similar to other vaccine stockpiles began being discussed in international meetings as the prospect of a low cost OCV (Shanchol®) obtaining WHO prequalification seemed likely [20]. During the same time, IVI began to develop an OCV demand forecast as a part of a global cholera investment case which was ultimately published in 2012 [21]. Through these estimates, it became apparent that new OCV suppliers were necessary to meet global demand as Shantha Biotechnics Ltd., was the only global manufacturer and had limited manufacturing capacity [21]. Moreover, for a healthy price competitive OCV market and to minimize supply risk more than one manufacturer is ideal.

To address this gap between demand and supply, IVI committed to increase the global OCV supply capacity by partnering with an additional manufacturer to develop, license and WHO prequalify an additional OCV. See time line of the Cholera Vaccine Program and development of OCVs in Table 1.

### 1.4 Increased global supply of affordable OCVs through partnership with a small bio-venture in South Korea

IVI reached out to several Developing Countries Vaccine Manufacturers (DCVMs) for technology transfer and product development partnerships, but most companies showed little interest, mainly because of limited commercial incentives. In 2009, IVI initiated discussions with a small South Korean bio-venture company (later named EuBiologics Co., Ltd) focusing on Contract Research and Manufacturing Organization (CRMO) services for the development of biologics. The company at that time did not have experience in vaccine production. However, their excellent capabilities, willingness to acquire vaccine production knowledge and commitment to make an impact on global public health resulted in a partnership with IVI, which started in September 2010.

The technology transfer of IVI’s process for the oral cholera vaccine and associated quality control, including lot release assays, began in October 2010. Production, quality control and quality assurance staff from EuBiologics spent time in the IVI laboratories and were given hands-on training in fermentation, downstream processing, and quality control, including LPS and toxin ELISA assays. By December 2010, a laboratory-scale (3 L) technology transfer was completed. These lots made at EuBiologics were evaluated by IVI scientists and deemed satisfactory. After the lab scale technology transfer IVI and EuBiologics initiated the research and development for fed batch production and in February 2011, EuBiologics successfully scaled up the process to manufacturing scale (30 L fermentation) and in December the same year to 100 L fermentation.

Preclinical toxicity studies were conducted in early 2011, and by September 2012, a phase I study was initiated by EuBiologics in Korea. In February 2013, the study was completed, confirming that their killed whole-cell OCV was safe, well-tolerated, and immunogenic [22].

In June the same year, a global cholera vaccine stockpile was established by WHO to ensure prompt availability of oral cholera vaccines and in December 2013, Gavi, the Vaccine Alliance announced a commitment of $115 million to fund the stockpile from 2014 to 2018. The Stockpile, initially created with 2 million doses of vaccine, and Gavi’s long term support of the stockpile, further reinforced the need for additional OCV producers like EuBiologics on the global market.

The phase I results enabled EuBiologics to proceed with a randomized controlled phase III trial to assess the safety and immunogenicity of their vaccine as compared to Shanchol®. This trial was conducted in the Philippines and immunogenicity testing was performed at the IVI labs in Seoul. The clinical study was completed in August 2014, and demonstrated that two doses of the EuBiologics’ vaccine induced vibriocidal responses comparable to those elicited by Shanchol® [23]. Based on these results, an export-only licensure application was submitted to Korea’s Ministry of Food and Drug Safety (MFDS) in September 2014, and in January 2015 the vaccine (Euvichol®) was approved.

In late January 2015, EuBiologics, with IVI support, submitted a dossier for WHO prequalification. By the end of the year, Euvichol® was WHO-prequalified, making it the second vaccine from the IVI pipeline to be WHO-prequalified. This formulation of Euvichol® was produced in a 100-liter fermenter, giving a production capacity of approximately 6 million doses per year. The company continued to make improvements to the vaccine. In 2014, EuBiologics increased production capacity by investing in a 600-liter fermenter and scaling up to enable production of up to 25 million doses per year by 2018. It also slightly changed the formulation by removing thimerosal, which was considered unnecessary in a single dose oral vaccine. This new variation of Euvichol® obtained WHO prequalification in September 2016.

**Fig. 3:**
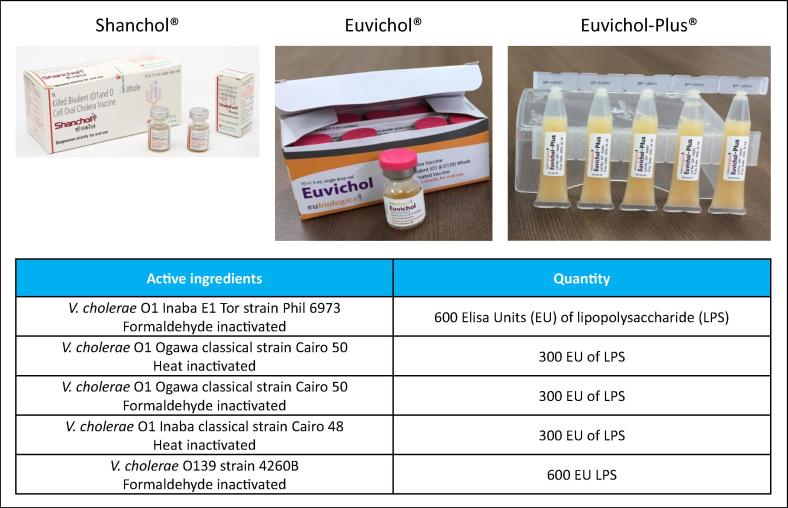
The WHO pre-qualified OCVs developed in collaboration between manufacturers and IVI. Detailed content and pictures of Shanchol™ (produced by Shantha Biotechnics Ltd., India), Euvichol® and Euvichol-Plus® (produced by EuBiologics Co., South Korea).

To further improve Euvichol®, EuBiologics recently changed the presentation of the vaccine from conventional glass vials to plastic tubes (Euvichol-Plus®, also thimerosal free), to facilitate delivery in emergency situations or humanitarian campaigns. The new plastic packaging of Euvichol-Plus® reduces the vial’s volume by nearly 30 percent and weight by over 50 percent, allowing easier transport and distribution of the vaccine and waste management. Compared with glass vials, the plastic packaging is also easier to open and administer. In addition, IVI recently started working with EuBiologics to obtain a Controlled Temperature Chain (CTC) [24] label for Euvichol-Plus®. The ability of OCVs to be kept outside traditional cold chain systems for limited periods in a controlled condition known as Controlled Temperature Chain, is important for its use in many outbreak/reactive and emergency vaccination settings. Shanchol™ achieved CTC label in February 2018 [25]. Euvichol-Plus® is currently available at $1.20 per dose. For detailed content and pictures of Shanchol™, Euvichol® and Euvichol-Plus® please see Fig. 3*.*

### 1.5 The role of an external not-for-profit organization in product development partnerships

#### 1.5.1 Reducing risks

From inception of the partnership between IVI and EuBiologics to WHO prequalification of Euvichol® and Euvichol-Plus®, it took 6 years and 10 months, and a total cost of approximately 19.7 million USD (including all costs for IVI and Eubiologics – please see Table 2 for breakdown of total cost by external funder). There were several factors that enabled this rapid and low-cost success. By transferring a vaccine that was already formulated and ready to be tested for safety and efficacy clinical trials, IVI reduced the risk of failure for EuBiologics and circumvented a long and possibly challenging clinical development phase. IVI’s previous experience and know-how in technology transfer enabled the development of a strong capacity among the EuBiologics staff in a short period of time. In addition, IVI helped EuBiologics accelerate the completion of the GMP-compliant manufacturing facility.

**Table 2:** Total costs for Euvichol broken down in percentage per external partners funders.

*Total expenditure by Eubiologics (17 Million USD)*	
Korea-Seoul Life Science Fund	20%
Green Cross Corporation	8%
Shinhan-K2 Investment Partners	6%
Global Health Investment Fund	29%
Korea Investment Global Frontier Fund	18%
Eubiologics	19%

Total expenditure by IVI (2.7 Million USD)*	
Bill & Melinda Gates Foundation	100%

* Does not include the unrestricted funding to IVI by i.e. the governments of Korea, Sweden and India.

To shorten regulatory timelines and reduce risk of delays, IVI and EuBiologics early in the partnership, initiated discussions with the Korean MFDS and the WHO-Prequalification group to set up a roadmap for licensure and pre-qualification of the product. The early initiation, and continuous discussions, enabled a fast and smooth licensure process, which reduced the time for the product to enter the global public market. Overall, early contact and continuous and productive discussion within the partnership and with agencies, external collaborators and funders have been vital components for the success.

#### 1.5.2 Securing financing and capital

Financing vaccine research and development is a major challenge, both for a vaccine PDP [26], [27] and for-profit vaccine development and production companies. The high cost for vaccine development often results in vaccine developers being reluctant to make the expensive and risky R&D investments needed to develop new vaccines.

In the case of the oral cholera vaccine, project funding was pooled from various sources of philanthropic and government support (e.g., Bill & Melinda Gates Foundation and the governments of Korea and Sweden) to IVI. As a nonprofit international organization recognized by Organization for Economic Co-operation and Development (OECD), IVI is eligible to receive international aid or Official Development Assistance (ODA). This funding was then leveraged to attract additional funding.

Acquiring the financing for clinical trials to test a vaccine against a neglected disease is a difficult proposition, particularly for a small local company with no previous track record in vaccine research and development. In this case, financial support was provided from donors through IVI to EuBiologics for the clinical development of Euvichol®. This was made possible due to the involvement of commercially neutral entities and philanthropic organizations like IVI and the Bill & Melinda Gates Foundation in the partnership, which signaled confidence and credibility in the vaccine and in the manufacturing partner. Their presence also gave EuBiologics international visibility, subsequently attracting investments by national and global health venture capitalists. Early in the collaboration, Korea-Seoul Life Science Fund (“KSLSF”), Green Cross Corporation and Shinhan-K2 Investment Partners – a Korean investment holdings company, invested in Eubiologics and, in 2014, the Global Health Investment Fund (GHIF) decided to make an investment in the company. Alongside KSLSF and Korea Investment Global Frontier Fund (“KIGFF”), GHIF supported the company with a total of approximately 5 million USD (2.5 million USD loan and 2.5 billion KRW investment) for capital equipment and support of the final clinical studies and regulatory preparations necessary to market Euvichol® to public-sector buyers worldwide (Table 2). These different economic investments were crucial for EuBiologics to make all the necessary investments in build-up of their production site, conduct the clinical studies and were all pivotal for getting the vaccine to the market.

#### 1.5.3 Creating commercial incentives

Limited commercial incentives often keep companies from investing in vaccine development for neglected diseases as they believe they will be unable to sell enough vaccine at a sufficient price to recoup their research costs. To help remove this obstacle, IVI facilitated relationships between EuBiologics and potential buyers of the vaccine (i.e. Gavi and UNICEF). These connections proved very important for the company and in 2016, EuBiologics signed a Long-Term Arrangement until 2018 with UNICEF to supply their oral cholera vaccine globally. IVI also provided resources and support to EuBiologics in developing an international commercialization and marketing strategy for Euvichol®.

### 1.6 The role of multiple stakeholders in generating evidence on OCV use and driving the demand

In addition to IVI’s efforts, multiple international not-for profit organizations, United Nations agencies, foundations and donors, universities and research institutes played a crucial role in generating evidence around the burden of cholera and the feasibility of OCV use, thus creating demand. Following the WHO prequalification of Shanchol™, more than 4.8 million doses were used between 2011 and 2015 in more than 21 campaigns conducted by various partners in India, Bangladesh, Haiti, Solomon Island, Thailand, Guinea, Sudan, Ethiopia, Malawi and Nepal, some countries deploying vaccine more than once [28], [29], [30], [31], [32], [33], [34], [35], [36], [37], [38], [39], [40], [41], [42], [43], [44]. These campaigns generated evidence of the feasibility, effectiveness and acceptability of vaccine in various endemic and epidemic settings including in humanitarian emergencies. The WHO prequalification of Euvichol® in 2015 greatly expanded the stockpile supply of OCV enabling more stockpile requests to be filled. From the initiation of the stockpile in 2013 through May 2018, around 25 million doses of OCV were deployed through campaigns in 19 different countries [45]. While the International Coordinating Group (ICG) reviewed and approved emergency stockpile requests, the Global Task Force on Cholera Control (GTFCC)[46], established in 2014 and with representation from over 15 partner organizations, has played a pivotal role in coordinating and managing stockpile requests for preventive campaigns and shaping international consensus on comprehensive cholera control. GTFCC partner organizations have continued to contribute to the body of evidence on cholera burden and control including use of OCV in alternative dosing regimens that provide flexibility and enhance feasibility [47], [48], [49], [50], [51], [52], [53], [54], [55], [56], [57]. As of 2018, the stockpile remains supply constrained with more requests for OCV then can be filled. As a result of the continued supply constraint, IVI is working with other potential manufacturers to become additional national or global suppliers.

### 1.7 Lessons learned

**Fig. 4:**
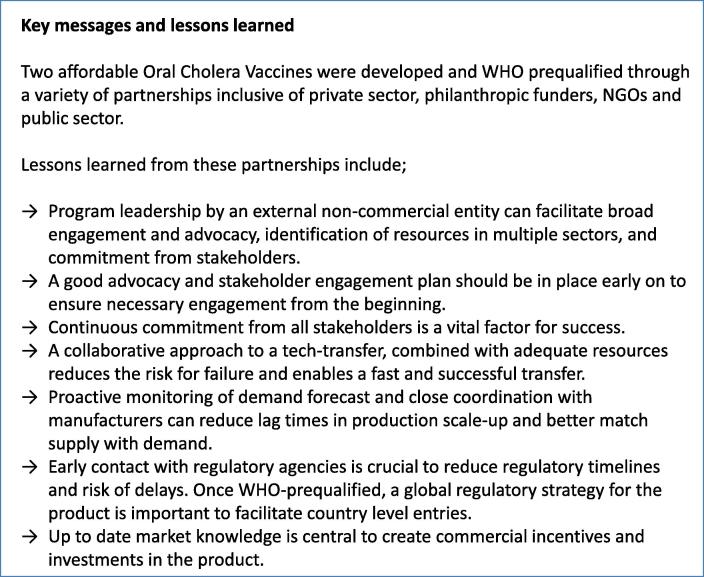
Key messages and lessons learned. Key messages and lessons learned through the development of affordable OCVs through a variety of partnerships inclusive of private sector, funders, NGOs and public sector.

Through the experience of the Cholera Vaccine program, many important lessons have been learned regarding what did work well but also what could have been done differently (please see Fig. 4 for a summary of the lessons learned). The major limitation early in the program was dependence on only one manufacturer. This caused constraint on the global supply of OCVs for a few years, which could have been avoided by doing additional technology transfers in parallel. In addition, the lack of a global regulatory strategy after WHO pre-qualification made the entry of OCV in various endemic countries challenging. It has also become evident how important it is to be clear with the manufacturing partners about demand forecast and anticipate the time lag to increase production capacity to avoid delay in supply. The uptake of OCV has exceeded early expectation due to the collective effort of many organizations, however it is important to recognize that the visibility of cholera as a disease of high mortality was magnified by large and tragic outbreaks such as occurred in Haiti. This large-scale tragedy led policymakers to move swiftly on OCV stockpile and financing. For future vaccine development efforts for global public health, it is important to have a strong advocacy and stakeholder engagement plan in place early on so as not to be reliant on coincident disaster to motivate action.

## 2 Conclusion

Developing and delivering vaccines for diseases mainly affecting people in developing countries is a difficult task due to the high costs and risks of vaccine R&D. IVI’s Cholera Vaccine Program, a philanthropic-funded, public-private partnership overcame the typical obstacles and brought to market affordable, safe, effective and easier-to-deliver vaccines against a neglected disease. However, in addition to generating a vaccine supply, multi-stakeholder engagement was necessary to generate essential evidence on the burden of disease, the feasibility, effectiveness and acceptability of use in multiple settings in order to create demand, conduct advocacy, and shape policy.

## References

[1] WHO. Global immunization data. February 2014; 2014.

[2] Cholera vaccines: WHO position paper - August 2017. Wkly Epidemiol Rec. 2017; 92(34): p. 477–98. 28845659

[3] Ali M., Nelson A.R., Lopez A.L., Sack D.A. (2015). Updated global burden of cholera in endemic countries. PLoS Negl Trop Dis.

[4] Shin S., Desai S.N., Sah B.K., Clemens J.D. (2011). Oral vaccines against cholera. Clin Infect Dis.

[5] Clemens J.D., Sack D.A., Harris J.R., Van Loon F., Chakraborty J., Ahmed F. (1990). Field trial of oral cholera vaccines in Bangladesh: results from three-year follow-up. Lancet.

[6] Sanchez J.L., Vasquez B., Begue R.E., Meza R., Castellares G., Cabezas C. (1994). Protective efficacy of oral whole-cell/recombinant-B-subunit cholera vaccine in Peruvian military recruits. Lancet.

[7] Cholera vaccines: WHO position paper. Wkly Epidemiol Rec. 2010; 85(13): p. 117–28. 20349546

[8] Anh D.D., Lopez A.L., Tran H.T., Cuong N.V., Thiem V.D., Ali M. (2014). Oral cholera vaccine development and use in Vietnam. PLoS Med.

[9] Trach D.D., Clemens J.D., Ke N.T., Thuy H.T., Son N.D., Canh D.G. (1997). Field trial of a locally produced, killed, oral cholera vaccine in Vietnam. Lancet.

[10] Trach D.D., Cam P.D., Ke N.T., Rao M.R., Dinh D., Hang P.V. (2002). Investigations into the safety and immunogenicity of a killed oral cholera vaccine developed in Viet Nam. Bull World Health Organ.

[11] Mahalanabis D., Lopez A.L., Sur D., Deen J., Manna B., Kanungo S. (2008). A randomized, placebo-controlled trial of the bivalent killed, whole-cell, oral cholera vaccine in adults and children in a cholera endemic area in Kolkata, India. PLoS ONE.

[12] Anh D.D., Canh D.G., Lopez A.L., Thiem V.D., Long P.T., Son N.H. (2007). Safety and immunogenicity of a reformulated Vietnamese bivalent killed, whole-cell, oral cholera vaccine in adults. Vaccine.

[13] Sur D., Lopez A.L., Kanungo S., Paisley A., Manna B., Ali M. (2009). Efficacy and safety of a modified killed-whole-cell oral cholera vaccine in India: an interim analysis of a cluster-randomised, double-blind, placebo-controlled trial. Lancet.

[14] Bhattacharya S.K., Sur D., Ali M., Kanungo S., You Y.A., Manna B. (2013). 5 year efficacy of a bivalent killed whole-cell oral cholera vaccine in Kolkata, India: a cluster-randomised, double-blind, placebo-controlled trial. Lancet Infect Dis.

[15] Sur D., Kanungo S., Sah B., Manna B., Ali M., Paisley A.M. (2011). Efficacy of a low-cost, inactivated whole-cell oral cholera vaccine: results from 3 years of follow-up of a randomized, controlled trial. PLoS Negl Trop Dis.

[16] Lucas M.E., Deen J.L., Von Seidlein L., Wang X.-Y., Ampuero J., Puri M. (2005). Effectiveness of mass oral cholera vaccination in Beira, Mozambique. New England J Med.

[17] Anh D.D., Lopez A.L., Thiem V.D., Long P.T., Son N.H., Deen J. (2007). Safety and immunogenicity of a reformulated Vietnamese bivalent killed, whole-cell, oral cholera vaccine in adults. Vaccine..

[18] Thiem V.D., Deen J.L., Von Seidlein L., Anh D.D., Park J.-K., Ali M. (2006). Long-term effectiveness against cholera of oral killed whole-cell vaccine produced in Vietnam. Vaccine.

[19] Cholera vaccines: WHO position paper. Weekly Epidemiological Record= Relevé épidémiologique hebdomadaire 2010; 85(13): p. 117–28. 20349546

[20] WHO. WHO Consultation on oral cholera vaccine (OCV) stockpile strategic framework: potential objectives and possible policy options: 18-20 September 2011, Geneva, Switzerland; 2012.

[21] Levin A., DeRoeck D., Kim Y., Clemens J., Lopez A., Ali M. (2012). An investment case for the accelerated introduction of oral cholera vaccines.

[22] Baik Y.O., Choi S.K., Kim J.W., Yang J.S., Kim I.Y., Kim C.W. (2014). Safety and immunogenicity assessment of an oral cholera vaccine through phase I clinical trial in Korea. J Korean Med Sci.

[23] Baik Y.O., Choi S.K., Olveda R.M., Espos R.A., Ligsay A.D., Montellano M.B. (2015). A randomized, non-inferiority trial comparing two bivalent killed, whole cell, oral cholera vaccines (Euvichol vs Shanchol) in the Philippines. Vaccine.

[24] WHO. Out of cold chain (OCC) and Controlled Temperature Chain (CTC) use of vaccines. Immunization Practices Advisory Committee; 2017 [updated 2017; cited]; http://www.who.int/immunization/programmes_systems/supply_chain/ctc/en/.

[25] WHO. WHO Prequalified Vaccines [cited]; Available from: https://extranet.who.int/gavi/PQ_Web/PreviewVaccine.aspx?nav=0&ID=249.

[26] Global Health Technologies Coalition (GHTC): Developing Technologies to Address Poverty-Related and Neglected Disease and Conditions (2013). Perspectives from nonprofits on accelerating product development and improving access for low- and middle-income countries.

[27] Increasing Access to Vaccines Through Technology Transfer and Local Production: WHO; 2011.

[28] Saha A., Chowdhury M.I., Khanam F., Bhuiyan M.S., Chowdhury F., Khan A.I. (2011). Safety and immunogenicity study of a killed bivalent (O1 and O139) whole-cell oral cholera vaccine Shanchol, in Bangladeshi adults and children as young as 1 year of age. Vaccine.

[29] Kar S.K., Sah B., Patnaik B., Kim Y.H., Kerketta A.S., Shin S. (2014). Mass vaccination with a new, less expensive oral cholera vaccine using public health infrastructure in India: the Odisha model. PLoS Negl Trop Dis.

[30] Ivers L.C., Teng J.E., Lascher J., Raymond M., Weigel J., Victor N. (2013). Use of oral cholera vaccine in Haiti: a rural demonstration project. Am J Trop Med Hygiene.

[31] Rouzier V., Severe K., Juste M.A.J., Peck M., Perodin C., Severe P. (2013). Cholera vaccination in urban Haiti. Am J Trop Med Hygiene.

[32] Luquero F.J., Grout L., Ciglenecki I., Sakoba K., Traore B., Heile M. (2014). Use of Vibrio cholerae vaccine in an outbreak in Guinea. N Engl J Med.

[33] Luquero F.J., Grout L., Ciglenecki I., Sakoba K., Traore B., Heile M. (2013). First outbreak response using an oral cholera vaccine in Africa: vaccine coverage, acceptability and surveillance of adverse events, Guinea, 2012. PLoS NeglTrop Dis.

[34] Ciglenecki I., Sakoba K., Luquero F.J., Heile M., Itama C., Mengel M. (2013). Feasibility of mass vaccination campaign with oral cholera vaccines in response to an outbreak in Guinea. PLoS Med.

[35] Porta M.I., Lenglet A., de Weerdt S., Crestani R., Sinke R., Jo Frawley M. (2014). Feasibility of a preventive mass vaccination campaign with two doses of oral cholera vaccine during a humanitarian emergency in South Sudan. Trans R Soc Trop Med Hyg.

[36] Phares C.R., Date K., Travers P., Déglise C., Wongjindanon N., Ortega L. (2016). Mass vaccination with a two-dose oral cholera vaccine in a long-standing refugee camp, Thailand. Vaccine.

[37] Tohme R.A., François J., Wannemuehler K., Iyengar P., Dismer A., Adrien P. (2015). Oral cholera vaccine coverage, barriers to vaccination, and adverse events following vaccination, Haiti, 2013. Emerg Infect Dis.

[38] WHO (2014). Oral cholera vaccine campaign among internally displaced persons in South Sudan. Weekly Epidemiological Record= Relevé épidémiologique hebdomadaire.

[39] Teshome S., Desai S., Kim J.H., Belay D., Mogasale V. (2018). Feasibility and costs of a targeted cholera vaccination campaign in Ethiopia. Human Vaccines Immunotherapeut.

[40] Msyamboza K.P., Hausi H., Chijuwa A., Nkukumila V., Kubwalo H.W., Im J. (2016). Feasibility and acceptability of oral cholera vaccine mass vaccination campaign in response to an outbreak and floods in Malawi. Pan African Med J.

[41] Kar S.K., Pach A., Sah B., Kerketta A.S., Patnaik B., Mogasale V. (2014). Uptake during an oral cholera vaccine pilot demonstration program, Odisha, India. Human Vaccines Immunotherapeut.

[42] Wierzba T.F., Kar S.K., Mogasale V.V., Kerketta A.S., You Y.A., Baral P. (2015). Effectiveness of an oral cholera vaccine campaign to prevent clinically-significant cholera in Odisha State, India. Vaccine.

[43] Burnett E., Dalipanda T., Ogaoga D., Gaiofa J., Jilini G., Halpin A. (2016). Knowledge, attitudes, and practices regarding diarrhea and cholera following an oral cholera vaccination campaign in the solomon islands. PLoS Negl Trop Dis.

[44] Hsiao A., Desai S.N., Mogasale V., Excler J.-L., Digilio L. (2017). Lessons learnt from 12 oral cholera vaccine campaigns in resource-poor settings. Bull World Health Organ.

[45] WHO. Oral Cholera Vaccine. [cited]; Available from: http://www.who.int/cholera/vaccines/en/.

[46] The Global Task Force on Cholera Control. [cited]; Available from: http://www.who.int/cholera/task_force/en/.

[47] Sauvageot D., Njanpop-Lafourcade B.-M., Akilimali L., Anne J.-C., Bidjada P., Bompangue D. (2016). Cholera incidence and mortality in Sub-Saharan African sites during multi-country surveillance. PLoS NeglTrop Dis.

[48] Lessler J., Moore S.M., Luquero F.J., McKay H.S., Grais R., Henkens M. (2018). Mapping the burden of cholera in sub-Saharan Africa and implications for control: an analysis of data across geographical scales. The Lancet.

[49] Bi Q., Ferreras E., Pezzoli L., Legros D., Ivers L.C., Date K. (2017). Protection against cholera from killed whole-cell oral cholera vaccines: a systematic review and meta-analysis. Lancet Infect Dis.

[50] Franke M.F., Ternier R., Jerome J.G., Matias W.R., Harris J.B., Ivers L.C. (2018). Long-term effectiveness of one and two doses of a killed, bivalent, whole-cell oral cholera vaccine in Haiti: an extended case-control study. Lancet Global Health.

[51] Azman A.S., Parker L.A., Rumunu J., Tadesse F., Grandesso F., Deng L.L. (2016). Effectiveness of one dose of oral cholera vaccine in response to an outbreak: a case-cohort study. Lancet Global Health.

[52] Qadri F., Ali M., Lynch J., Chowdhury F., Khan A.I., Wierzba T.F. (2018). Efficacy of a single-dose regimen of inactivated whole-cell oral cholera vaccine: results from 2 years of follow-up of a randomised trial. Lancet Infect Dis.

[53] Ferreras E., Chizema-Kawesha E., Blake A., Chewe O., Mwaba J., Zulu G. (2018). Single-dose cholera vaccine in response to an outbreak in Zambia. N Engl J Med.

[54] Qadri F., Wierzba T.F., Ali M., Chowdhury F., Khan A.I., Saha A. (2016). Efficacy of a single-dose, inactivated oral cholera vaccine in Bangladesh. N Engl J Med.

[55] Poncin M., Zulu G., Voute C., Ferreras E., Muleya C.M., Malama K. (2018). Implementation research: reactive mass vaccination with single-dose oral cholera vaccine, Zambia. Bull World Health Organ.

[56] Kanungo S., Desai S.N., Nandy R.K., Bhattacharya M.K., Kim D.R., Sinha A. (2015). Flexibility of oral cholera vaccine dosing—a randomized controlled trial measuring immune responses following alternative vaccination schedules in a cholera hyper-endemic zone. PLoS NeglTrop Dis.

[57] Sauvageot D., Saussier C., Gobeze A., Chipeta S., Mhango I., Kawalazira G. (2017). Oral cholera vaccine coverage in hard-to-reach fishermen communities after two mass Campaigns, Malawi, 2016. Vaccine.

[58] Milstien J., Cohen J.C., Olsen I.T. (2007). An evaluation of GAVI Alliance efforts to introduce new vaccines via the Accelerated Development and Introduction Plans (ADIPs) and the Hib Initiative (HI).

